# Universal field-tunable terahertz emission by ultrafast photoinduced demagnetization in Fe, Ni, and Co ferromagnetic films

**DOI:** 10.1038/s41598-020-72855-1

**Published:** 2020-09-28

**Authors:** Lin Huang, Sang-Hyuk Lee, Seon-Dae Kim, Je-Ho Shim, Hee Jun Shin, Seongheun Kim, Jaehun Park, Seung-Young Park, Yeon Suk Choi, Hyun-Joong Kim, Jung-Il Hong, Dong Eon Kim, Dong-Hyun Kim

**Affiliations:** 1 Department of Physics, Chungbuk National University, Cheongju, 28644 South Korea; 2 Department of Physics and Center for Attosecond Science and Technology, POSTECH, Pohang, 37673 South Korea; 3 Max Planck POSTECH/KOREA Research Initiative, Pohang, 37673 South Korea; 4 Pohang Accelerator Laboratory, POSTECH, Pohang, 37673 South Korea; 5 Center for Scientific Instrumentation, Korea Basic Science Institute, Daejeon, 34133 South Korea; 6 Department of Emerging Materials Science, Daegu Gyeongbuk Institute of Science and Technology, Daegu, 42988 South Korea

**Keywords:** Magneto-optics, Terahertz optics, Ultrafast photonics

## Abstract

We report a universal terahertz (THz) emission behavior from simple Ni, Fe, and Co metallic ferromagnetic films, triggered by the femtosecond laser pulse and subsequent photoinduced demagnetization on an ultrafast time scale. THz emission behavior in ferromagnetic films is found to be consistent with initial magnetization states controlled by external fields, where the hysteresis of the maximal THz emission signal is observed to be well-matched with the magnetic hysteresis curve. It is experimentally demonstrated that the ultrafast THz emission by the photoinduced demagnetization is controllable in a simple way by external fields as well as pump fluences.

## Introduction

Very recently, THz spintronics in ferromagnetic materials has attracted much attention due to possible future applications^1,2^. THz spintronics based on an interaction between THz wave and spins could be promising since it is involved with key aspects of spintronics such as spin transport^3,4^, spin wave excitation^5^, spin current control^6^, and spin-flop transition^7^.

On the other hand, THz emission from the ferromagnetic materials has been explored as well since the seminal work on a femtosecond laser-induced demagnetization dynamics in Ni film^8^, followed by numerous extensive studies on photoinduced ultrafast spin dynamics^9–15^. One of the important finding regarding the ultrafast photoinduced demagnetization is that THz light should be emitted from ferromagnetic thin films when the film is illuminated by femtosecond laser pulses so that the subsequent photoinduced demagnetization/remagnetization generates the THz emission^15^. However, relatively few studies have been reported for THz emission by ultrafast demagnetization. Moreover, there have been reports that there might be other THz emission mechanisms such as an inverse spin Hall effect in addition to the ultrafast demagnetization^16,17^. Recently, we have reported our direct observation of THz emission and photoinduced demagnetization/remagnetization signals in Co films with comparison to each other, demonstrating that the THz emission from the ultrafast demagnetization becomes dominant in relatively thick (> 30 nm) Co films, regardless of selection of capping layers^18^. For THz emission by inverse spin Hall effect, a strong spin–orbit coupling occurring in interface between ferromagnetic and heavy metal layers is required as in Fe/Pt^19^, Fe/Au, and Fe/Ru^6^ bilayers where the emission behavior sensitively depends on the film thickness and capping layer selection^6,18,20^. Thus, to realize a THz spintronics, THz emission behavior requires a further understanding. Unfortunately, little studies have been devoted to a field-dependent THz emission probably due to several emission mechanisms and sensitivity to the material selection, although systematic investigation on the field-dependent THz emission behavior could be the first step for applications. For Ta/CoFeB/MgO trilayer films, a field-dependent THz emission by the inverse spin Hall effect has been reported^21^. Recently, THz emission by the ultrafast demagnetization of 20-nm NdFeCo and GdFeCo films together with a 12-nm Co film with oppositely saturated magnetization^22^ has been reported, where the measured THz emissions from GdFeCo and NdFeCo alloy films are found to have a faster magnetization dynamics due to Nd element. Thus, a systematic study of THz emission from the ultrafast demagnetization dynamics with variation of element as well as external magnetic fields becomes necessary. Here, we report our experimental investigation on the field-dependent THz emission by ultrafast photoinduced demagnetization in 30-nm Fe, Ni, and Co films with variation of external fields, where a universal THz emission behavior is regardless of film elements is clearly observed with field-tunability.

## Results

**Figure 1:**
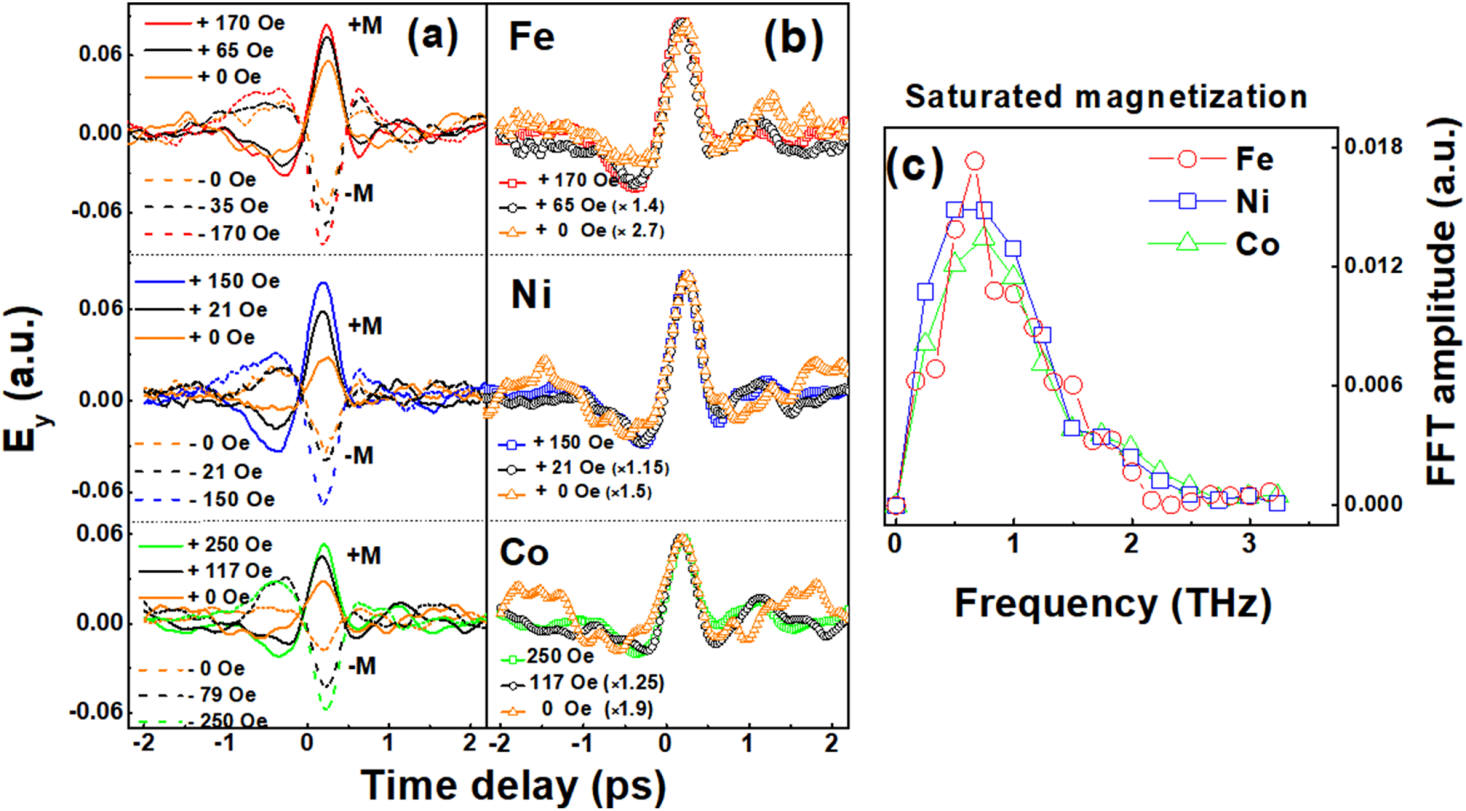
(**a**) THz emission profiles from Ni, Fe, and Co 30 nm films under several external fields. (**b**) The curves represent THz signals E_y_ from Fe, Ni and Co compare to the saturated field state. (**c**) Fourier transformed spectra of the THz emission signals for the cases of + *M* and *– M*.

The electric field profile of emitted THz wave upon ultrafast demagnetization of Fe, Ni, and Co films is illustrated in Fig. 1a for several magnetic states including oppositely saturated states. It is clear that the THz emission profile of Co film (bottom) from oppositely saturated states are quite symmetric, as reported in^22^. As seen in the figure, it seems that the THz emission profile is not significantly different for each element. It is also observed that peak amplitudes of generated THz wave are gradually changing by external magnetic fields for all cases of Fe, Ni, and Co thin films, allowing a control of the THz emission strength easily by external fields. The overall profile and field-dependency does not seem to be depending on the element selection, implying that the THz emission could be simply originated from the ultrafast photoinduced demagnetization of Fe, Ni, and Co films. It should be mentioned that there have been reports on different timescales for Fe, Ni, and Co elements^12,13,24–29^. We consider that there have been substantial differences in timescale with respect to measurement technique and sample thickness. The capping layer role particularly for thinner samples should be also seriously taken into consideration. Moreover, there has been a report about the difference in ultrafast demagnetization for magnetic moment either from intrinsic one or induced one^13^, which could add complexity. In this regard, we believe that our measurement makes sense because we compared for single-elemental thick (30 nm) film of Co, Ni, and Fe with the same Pd capping layer, expecting the dominant THz emission mechanism to be involved with ultrafast demagnetization, thereby hoping to minimize the complex effect of ferromagnetic element with capping layer in observed THz signal.

In Fig. 1b, THz emission waves at non-saturated intermediate magnetic states are compared to the saturated one with proper normalization multipliers denoted in the figure, where a shape of all data matches with one another without a particular dependence on the external field strength. It should be also noted that Fourier transformed (FT) signals of the THz emissions from 3 different elements are almost the same as shown in Fig. 1c. For comparison, FT of THz emissions from negatively saturated states of 3 elements are plotted. The frequency range in Fig. 1c roughly matches but with substantial difference when compared to previous experimental reports for Co (0–3 THz)^22^, Fe (0–1.5 THz)^23^, and Ni (0.5–4 THz)^29^ films. It should be commented that the seemingly universal THz emission behavior observed in the present study regardless of element selection is observable after selecting thick enough (~ 30 nm) films based on our previous work^18^ to exactly distinguish the THz emission contribution by the ultrafast photoinduced demagnetization dynamics from other possible mechanisms originated from the interface.

**Figure 2:**
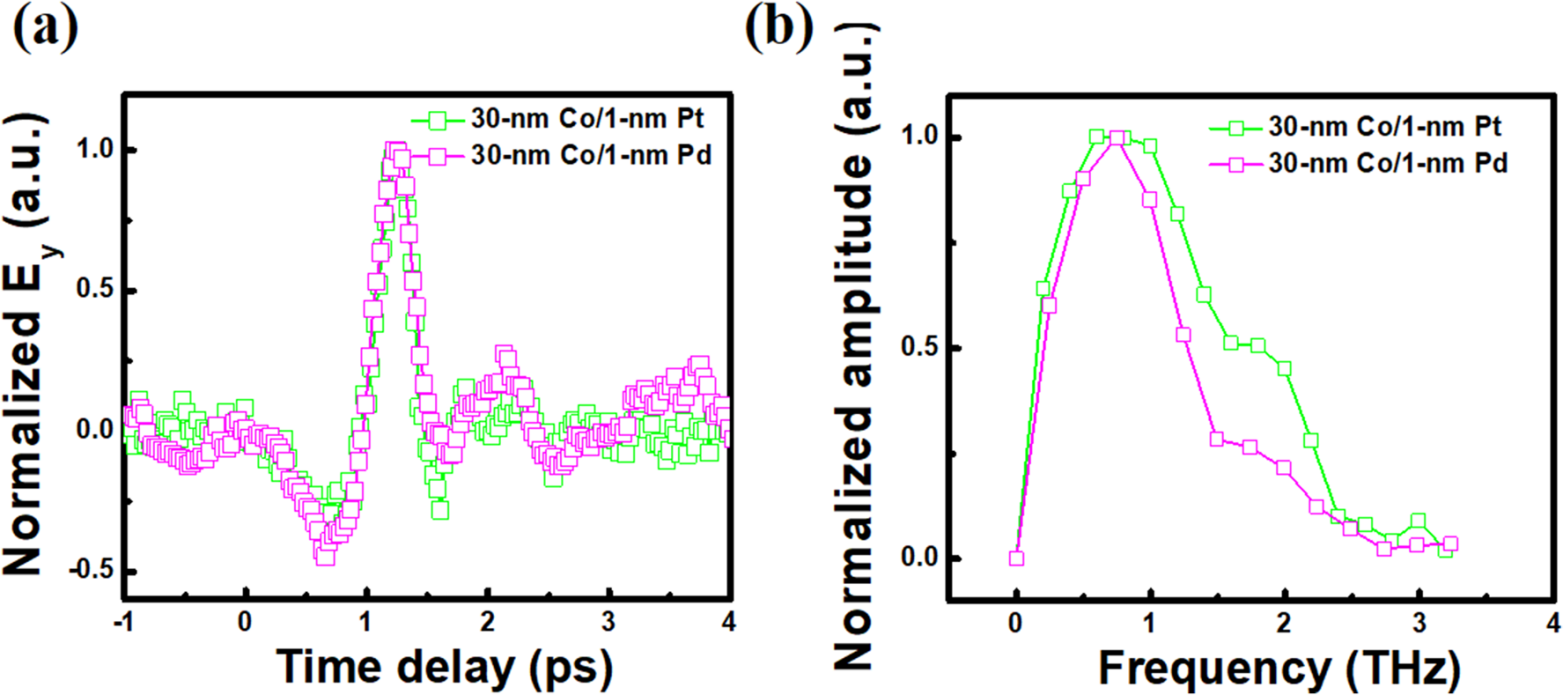
Comparison of (**a**) THz emission profiles and (**b**) FFT spectra for 30-nm Co with 1-nm Pt or 1-nm Pd capping layer at a positively saturated state.

We have also directly compared the THz emission signals from 30-nm Co with 1-nm Pt^18^ and with 1-nm Pd (present work), as in Fig. 2a, where we could see no significant difference in the emission profiles. The FFT spectra of the THz emission for two cases with Pt and Pd capping layers are plotted in Fig. 2b. The major peak regions in the spectra are roughly matching although there is a little deviation which could be originated from different capping layer role. It is interesting to note that the difference in THz emission spectra by selecting different element among Co, Fe, and Ni seems to be smaller than the difference by selecting different capping layer between Pt and Pd.

**Figure 3:**
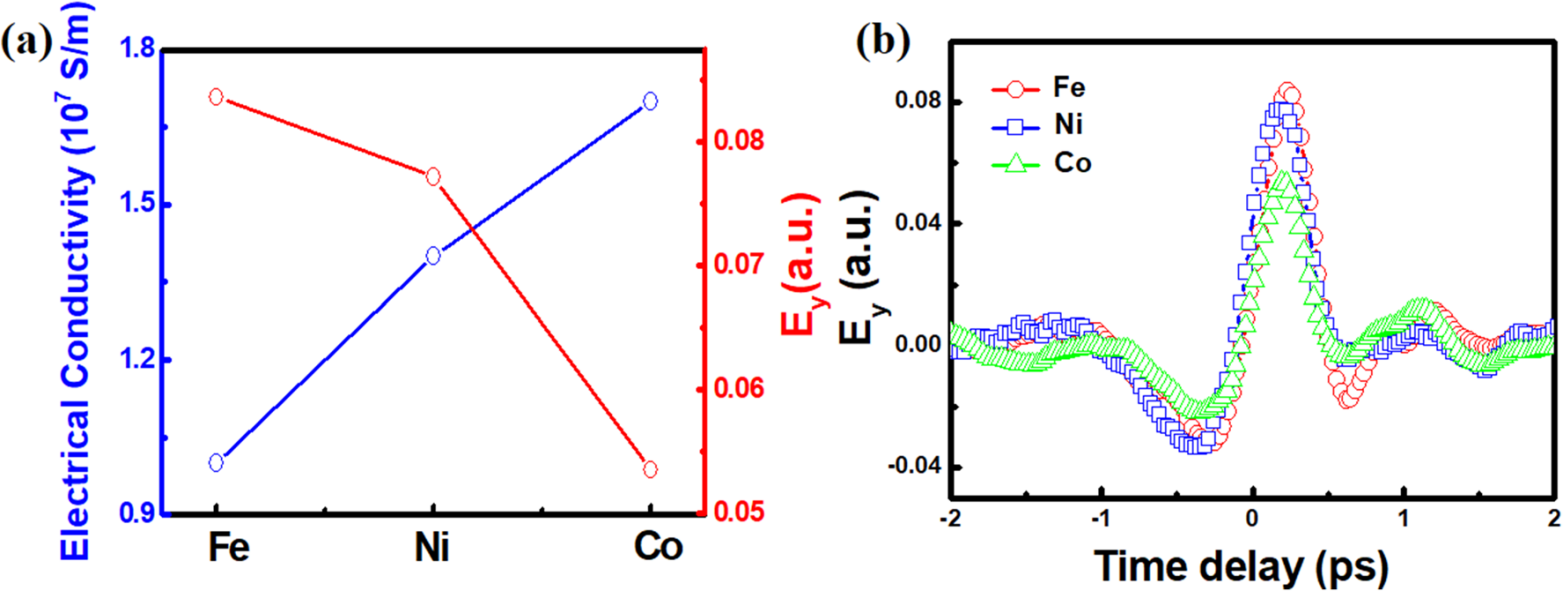
(**a**) The electrical conductivity σ and the maximum amplitude of THz signal E_y_ max for Ni, Fe, and Co 30 nm films. (**b**) Comparison of THz emission profiles for Ni, Fe, and Co films at a positively saturated state.

As a pump laser irradiate on the sample surface to induce the ultrafast demagnetization dynamics, THz wave emitted from a sample should pass through the sample to be detected in the end. If a current density *J* and the conductivity *σ* are approximated to follow the Ohm’s law in metallic films of Fe, Ni, and Co^30^: the electric field E is described as: $E \approx \frac{J}{\sigma }$. In other word, the amplitude of THz wave is expected to be inversely proportional to the conductivity *σ* of metallic samples, which is demonstrated in Fig. 3a. *σ* value of each film is from the literature values^31,32^. It is clearly observed that the amplitude of electric field profile (E_y_ max) is inversely proportional to *σ* for Fe, Ni and Co. Electric field profiles of emitted THz wave at positively saturated magnetic state for Fe, Ni, and Co are plotted in Fig. 3b, where the lowest (highest) amplitude is observed for Co (Fe) with highest (lowest) conductivity.

**Figure 4:**
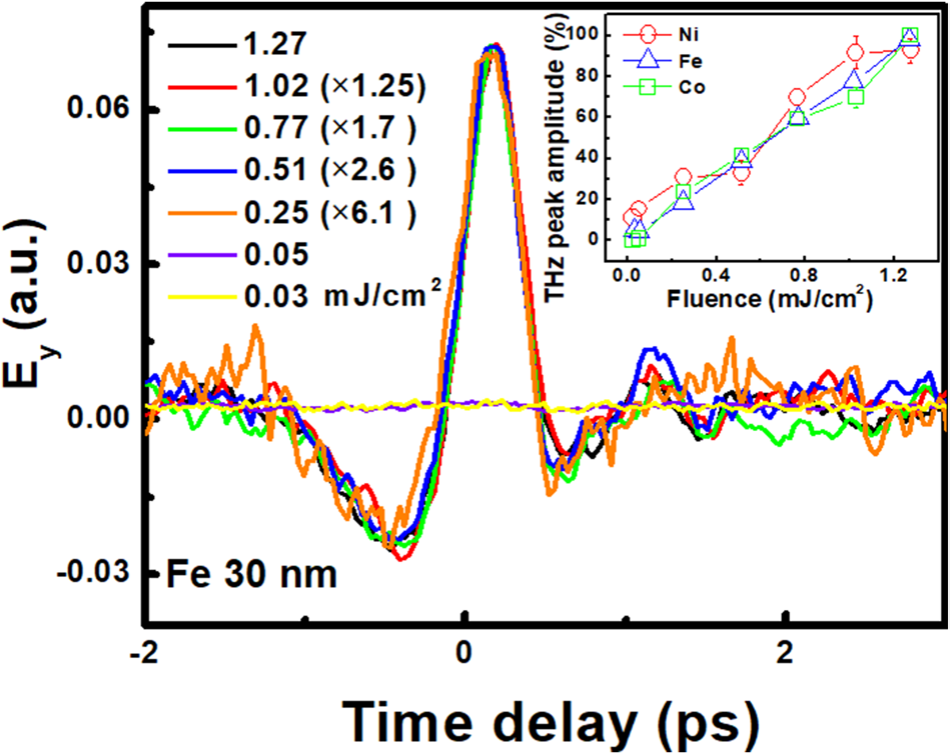
THz emission from Fe film with various fluences from 0.03 to 1.27 mJ*/*cm^2^. Inset figure is THz peak amplitude for Ni (open circle), Fe (open triangle), and Co (open square) thin films with respect to the fluences.

Since THz emission is dominantly contributed from the ultrafast photoinduced demagnetization, it is expected to have stronger THz emission by the pump pulse with higher fluences due to larger demagnetization. THz emission profile with various fluences is plotted in Fig. 4 for the case of Fe film. It is observed that there is no significant change in terms of shape and phase of *E*_*y*_ after normalization by multiplying proper numbers, whereas amplitudes gradually increase with respect to the pump fluence. The same trend is also found for Ni and Co films as well. The amplitude of THz emission with variation of pump fluences are shown for Fe, Ni, and Co films in the inset figure, where it is clearly observed that the THz amplitude increases as the fluence increases. It should be also noted that the THz peak amplitude variation with respect to the pump fluences for different 3 elements again falls into the same overall tendency when the signal is normalized by the maximum peak intensity of each element. It is clearly observed that the THz amplitude increases as fluence increases.

**Figure 5:**
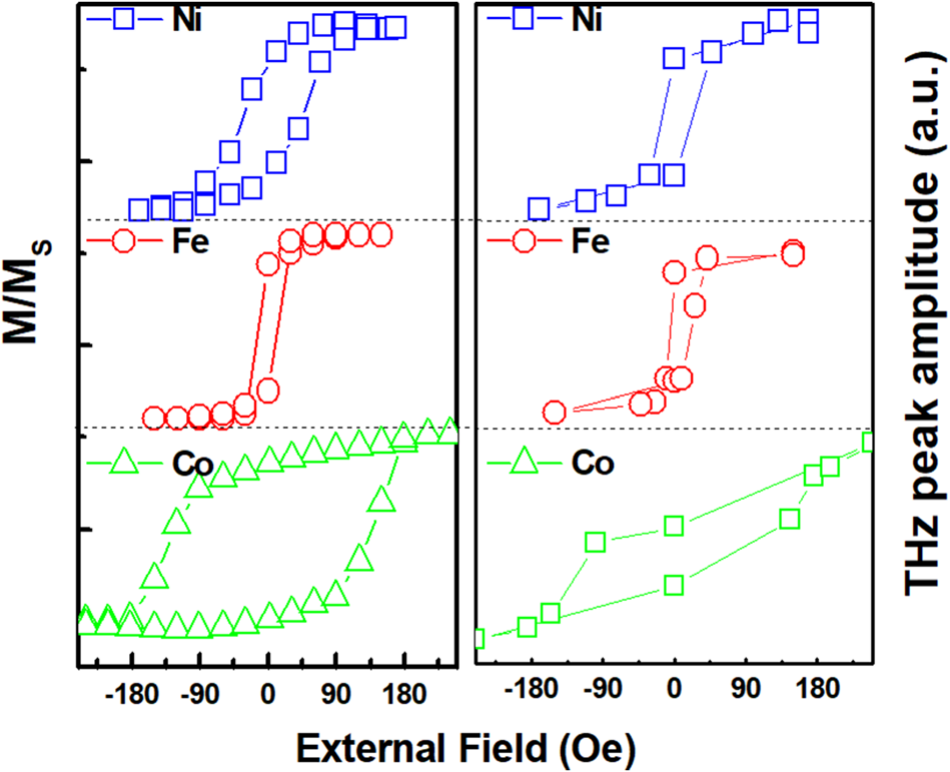
(**a**) Magnetic hysteresis loops of Ni (open square), Fe (open circle), and Co (open triangle) films along the in-plane direction. (**b**) Hysteresis curves of THz peak amplitude under the same cycling external fields.

We have further analyzed THz emission behavior with variation of external fields, as seen in Fig. 5. Hysteresis curves measured for Fe, Ni, and Co films are plotted in Fig. 5a, while THz peak amplitude measured with respect to the field strength is plotted in Fig. 5b. It is very interesting to note that there also exists a hysterectic behavior even in THz emission, which is a clear indication that the THz emission is basically from the magnetic state of ferromagnetic films. This is explainable since the stroboscopic observation of THz emission, originated from the ultrafast photoinduced demagnetization, should depend on the initial magnetization state along the magnetic hysteresis curve of Fe, Ni, and Co films. Correlated hysteresis curves of magnetization and THz emission peak amplitude under cycling external fields implies a direct proportional control of the THz emission behavior based on the magnetization state information on an ultrafast time scale. Since the polarity of emitted THz wave depends exactly on the initial magnetization direction (Fig. 1a), it is concluded that the emitted THz peak amplitude can be fully tunable within the range spanned by the magnetization state along the whole magnetic hysteresis curve.

## Discussion

In conclusion, we have explored the field-dependent THz emission in 30 nm Fe, Ni, and Co films, where ultrafast demagnetization universally generates THz emission regardless of elements. It is observed that the THz emission behavior directly corresponds to the magnetization state of samples, where field-tunability as well as fluence-tunability of the ultrafast THz emission on a femtosecond time scale is experimentally demonstrated. Our finding is believed to be an important experimental evidence in designing future THz spintronic devices operating on an ultrafast time scale.

## Methods

Fe, Ni, and Co films were prepared by sputtering on SiO_2_ substrate with thickness to be all fixed to be 30 nm. The 30-nm thickness was selected since it has been confirmed that dominant THz emission mechanism in 30-nm Co film is the photoinduced ultrafast demagnetization/remagnetization dynamics^18^ and that the effect of other mechanisms such as inverse spin Hall effect, arising from the interface, can be neglected. On top of Fe, Ni, and Co layer, 1-nm Pd was deposited as a capping layer. Stroboscopic optical pump and THz emission measurement was carried out at the fs-THz beamline of Pohang Accelerator Laboratory, as the overall optical layout is illustrated in Ref.^33^. Ti:Sapphire regenerative amplifier was used to produce a laser pulse of 800 nm wavelength with 1 kHz repetition rate, 3.2 W power, and 120 fs duration. THz radiation emitted from the sample is measured by electro-optic (EO) sampling method. THz radiation is collected and focused onto a 10 × 10 mm^2^ $\left\langle {110} \right\rangle$ ZnTe nonlinear crystal (3-mm thickness) using parabolic mirrors, which can be measured by a combination of a quarter-wave plate, a Wollaston polarizer, and a pair of balanced photodiodes. The signal current was sent to a lock-in amplifier. THz low pass filter (Microtech, Inc.) was used to remove the residual pump beam. Spectral bandwidth of the detector is found to cover up to 2.5 THz. Arbitrary unit is used in the THz emission signal due to the low signal intensity for calibration using other detectors such as bolometer or pyroelectric detector. We estimate that THz field in the present study might be lower than 1 kV/cm. The fluence of pump pulse was varied from 0.03 to 1.27 mJ/cm^2^ (300 μJ, area: ~ 0.2 cm^2^) with keeping the pump beam size to be 5 mm. In-plane external magnetic field was applied to film samples to control the magnetic states along the full hysteresis curve with the maximum field of 250 Oe.

## Data Availability

The data that support the findings of this study are available from the corresponding authors upon reasonable request.
